# Intestinal Damage and Inflammatory Biomarkers in Human Immunodeficiency Virus (HIV)–Exposed and HIV-Infected Zimbabwean Infants

**DOI:** 10.1093/infdis/jix367

**Published:** 2017-07-28

**Authors:** Andrew J Prendergast, Bernard Chasekwa, Sandra Rukobo, Margaret Govha, Kuda Mutasa, Robert Ntozini, Jean H Humphrey

**Affiliations:** 1 Zvitambo Institute for Maternal and Child Health Research, Harare, Zimbabwe;; 2 Department of International Health, Johns Hopkins Bloomberg School of Public Health, Baltimore, Maryland; and; 3 Blizard Institute, Queen Mary University of London, United Kingdom

**Keywords:** HIV, infant, Africa, inflammation, mortality, intestinal

## Abstract

**Background:**

Disease progression is rapid in human immunodeficiency virus (HIV)–infected infants. Whether intestinal damage and inflammation underlie mortality is unknown.

**Methods:**

We measured plasma intestinal fatty acid binding protein (I-FABP), soluble CD14 (sCD14), interleukin 6 (IL-6), and C-reactive protein (CRP) at 6 weeks and 6 months of age in 272 HIV-infected infants who either died (cases) or survived (controls), and in 194 HIV-exposed uninfected (HEU) and 197 HIV-unexposed infants. We estimated multivariable odds ratios for mortality and postnatal HIV transmission for each biomarker using logistic regression.

**Results:**

At 6 weeks, HIV-infected infants had higher sCD14 and IL-6 but lower I-FABP than HIV-exposed and HIV-unexposed infants (*P* < .001). CRP was higher in HIV-exposed than HIV-unexposed infants (*P* = .02). At 6 months, HIV-infected infants had highest sCD14, IL-6, and CRP concentrations (*P* < .001) and marginally higher I-FABP than other groups (*P* = .07). CRP remained higher in HIV-exposed vs HIV-unexposed infants (*P* = .04). No biomarker was associated with mortality in HIV-infected infants, or with odds of breast-milk HIV transmission in HIV-exposed infants.

**Conclusions:**

HIV-infected infants have elevated inflammatory markers by 6 weeks of age, which increase over time. In contrast to adults and older children, inflammatory biomarkers were not associated with mortality. HEU infants have higher inflammation than HIV-unexposed infants until at least 6 months, which may contribute to poor health outcomes.

## Summary

HIV-infected Zimbabwean infants had high levels of inflammation, but no biomarker was associated with mortality. HIV-exposed but uninfected infants had higher C-reactive protein than HIV-unexposed infants, but baseline levels of intestinal damage were not related to subsequent HIV acquisition through breastfeeding.

Despite improved coverage of prevention of mother-to-child transmission (PMTCT) interventions, approximately 150000 infants acquired human immunodeficiency virus (HIV) in 2015 [1]. Mortality among HIV-infected infants exceeds 50% by 2 years in sub-Saharan Africa without antiretroviral therapy (ART) [2]. The reasons for rapid disease progression are incompletely understood [3], although advanced maternal disease and high infant viral loads contribute [4–7].

Immune activation is a hallmark of HIV, likely driven by viral replication, coinfections, and microbial translocation [8]. In adults, the intestinal tract is targeted early in acute infection [9], with profound loss of CD4 cells [10, 11] and epithelial damage [12] leading to impaired intestinal barrier function [9] and microbial translocation, which may contribute to immune activation [13]. In adults, pre-ART levels of inflammatory biomarkers predict progression to AIDS and death [14–19].

In a recent study of HIV-infected children in Uganda and Zimbabwe [20], pre-ART levels of inflammatory biomarkers (interleukin [IL-6] or C-reactive protein [CRP]) predicted mortality independently of CD4 count. Whether intestinal damage, microbial translocation, and immune activation are associated with mortality during infancy (first year of life) remains uncertain. Mortality peaks around 2–6 months of age following perinatal transmission and remains high throughout infancy [21, 22]. Although elevated lipopolysaccharide and soluble inflammatory biomarkers have been reported [23–28], no studies have assessed associations with mortality during infancy.

As PMTCT coverage improves, there is a growing population of HIV-exposed uninfected (HEU) infants, who have increased morbidity and mortality compared with HIV-unexposed infants [29] and remain at risk of infection through breastfeeding [30]. There is a need to characterize the immune milieu in HEU infants [31] and to understand the contribution of intestinal damage and immune activation to postnatal HIV acquisition [29].

We used archived samples from a birth cohort of Zimbabwean infants to evaluate biomarkers of intestinal damage and inflammation. Our hypotheses were, first, that HIV-infected infants who died would have higher biomarker levels than those who survived; second, that HIV-exposed infants would have higher biomarker levels than HIV-unexposed infants; and, third, that HEU infants who subsequently acquire HIV through breastfeeding would have higher baseline biomarker levels than those remaining uninfected.

## METHODS

### ZVITAMBO Trial

This study used data and samples from the Zimbabwe Vitamin A for Mothers and Babies (ZVITAMBO) trial, conducted in Zimbabwe between 1997 and 2001 [32, 33]. A total of 14110 mother–infant pairs were recruited within 96 hours of delivery following written informed consent, provided neither had an acute life-threatening condition, the infant was a singleton with birthweight ≥1500 g, and the mother planned to stay in Harare. Mothers and infants were randomized to single-dose vitamin A or placebo, and followed at 6 weeks, 3 months, then every 3 months in a study clinic as previously described [32, 33]. Data on feeding practices at 3 months were used to categorize exclusive, predominant, or mixed breastfeeding [34]. Anthropometry was undertaken at each visit using standardized techniques [35]. The trial was conducted prior to introduction of cotrimoxazole prophylaxis or ART in Zimbabwe. Infant mortality was ascertained up to 12 months for all infants and up to 24 months for a subset [22].

### Sample Collection and HIV Testing

Blood was collected from mothers and infants at baseline and from all HIV-exposed infants and a representative subsample of HIV-unexposed infants at each visit. Plasma was stored in –80°C freezers with automatic generator backup. At recruitment, mothers were tested for HIV using 2 parallel enzyme-linked immunosorbent assays (ELISAs) (HIV 1.0.2 ICE, Murex Diagnostics; GeneScreen HIV 1/2, Sanofi Diagnostics Pasteur). Infant HIV status was ascertained retrospectively by HIV serology (GeneScreen ELISA) if ≥18 months, or DNA polymerase chain reaction (PCR) (Roche Amplicor version 1.5, Roche Diagnostic Systems, Alameda, California) if <18 months. Infants testing HIV DNA PCR positive at baseline (within 96 hours of birth) were classified as intrauterine infected; infants testing HIV DNA PCR negative at baseline but positive at 6 weeks were classified as intrapartum infected; infants testing HIV DNA PCR negative at 6 weeks but positive subsequently were classified as postnatally infected. Infants were censored at their last HIV test result. Viral load was measured in HIV-infected infants with available plasma at 6 weeks [6].

### Selection of Study Subjects

#### HIV-Infected Infants

**Figure 1.**
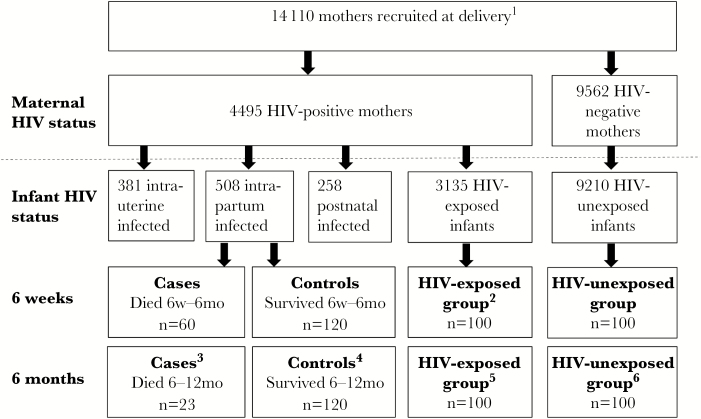
Selection of human immunodeficiency virus (HIV)–infected, HIV-exposed, and HIV-unexposed infants. A total of 14110 mother–infant pairs were recruited to the ZVITAMBO trial within 96 hours of delivery. Maternal HIV testing identified 4495 HIV-positive and 9562 HIV-negative women at baseline; 53 women had unknown HIV status. From 508 intrapartum-infected infants (HIV DNA polymerase chain reaction negative at birth and positive at 6 weeks), we selected cases and controls for the current study. At 6 weeks, cases were intrapartum-infected infants who subsequently died between 6 weeks and 6 months of age; controls were intrapartum-infected infants who survived to 6 months. At 6 months, cases were intrapartum-infected infants who subsequently died between 6 and 12 months of age; controls were intrapartum-infected infants who survived to 12 months. At 6 weeks and 6 months, we randomly selected 100 HIV-unexposed infants with available plasma samples, and purposively selected 100 HIV-exposed infants with available plasma samples who either did or did not subsequently acquire postnatal HIV through breastfeeding. ^1^Fifty-three women had unknown HIV status. ^2^Forty-five infants subsequently acquired HIV through breastfeeding between 6 weeks and 6 months and 55 remained uninfected. ^3^Six HIV-infected controls from the 6-week time-point were cases at the 6-month time-point. ^4^45 HIV-infected controls from the 6-week time-point were also selected as controls at the 6-month time-point. ^5^Six HIV-exposed infants from the 6-week time-point were also selected at the 6-month time-point. Of these 100 infants, 50 infants subsequently acquired HIV through breastfeeding between 6 and 12 months and 50 remained uninfected. ^6^Three HIV-unexposed infants from the 6-week time-point were also selected at the 6-month time-point.

We conducted a case-control study of HIV-infected infants to evaluate associations between plasma biomarkers and mortality in the first half of infancy (for 6-week biomarkers) or second half of infancy (for 6-month biomarkers) (Figure 1). We restricted analysis to intrapartum-infected infants, because we previously showed that timing of transmission was a determinant of mortality [22]. At 6 weeks, we selected intrapartum-infected infants who died (cases) or survived (controls) between 6 weeks and 6 months, and had sufficient cryopreserved plasma available. At 6 months of age, we selected intrapartum-infected infants who died (cases) or survived (controls) between 6 and 12 months, and had sufficient cryopreserved plasma available. Infants who were controls at 6 weeks could be selected as cases or controls at 6 months. Causes of death were assigned by a study pediatrician as previously reported [22].

#### HIV-Unexposed and HIV-Exposed Infants

At 6 weeks and 6 months we selected HIV-unexposed and HIV-exposed infants to provide comparative biomarker data (Figure 1). HIV-exposed infants were defined as infants born to a mother testing HIV positive at delivery who did not acquire intrauterine or intrapartum HIV; we purposively selected infants who subsequently did or did not acquire HIV through breastfeeding and had sufficient cryopreserved plasma available, to compare baseline biomarkers between groups. HIV-unexposed infants were selected randomly from the pool of infants born to mothers testing HIV negative at delivery and who did not subsequently seroconvert, provided the infant had sufficient cryopreserved plasma available. Infants selected at 6 weeks could also be selected at 6 months.

### Sample Size Calculation

We used data from HIV-positive adults, in whom baseline plasma soluble CD14 (sCD14) levels independently predicted mortality [36], to calculate our sample size. At each time-point, we aimed to select 60 HIV-infected cases (died) and 120 HIV-infected controls (survived), giving >80% power to detect an odds ratio (OR) of 2.7 for the association between elevated sCD14 and mortality, assuming 20%–25% of controls had elevated sCD14, or 80% power to detect an OR of 3.0, assuming 15% controls had elevated sCD14. All ORs were conservative estimates compared to the effect size reported in adults [36]. We had no data to inform a sample size for comparison groups, so chose 100 HIV-exposed and 100 HIV-unexposed infants at each time-point to provide normative biomarker data.

### Measurement of Biomarkers

We measured biomarkers of small intestinal damage (intestinal fatty acid binding protein [I-FABP]; Hycult Biotechnology, Uden, the Netherlands), monocyte activation (sCD14; R&D Systems, Minneapolis, Minnesota) and inflammation (IL-6 and CRP; both R&D Systems) by ELISA in cryopreserved plasma, according to manufacturers’ instructions. Biomarkers were selected based on their associations with mortality in prior studies [14–20, 36].

### Statistical Analysis

Baseline characteristics and plasma biomarkers were compared between groups using χ^2^ tests for categorical variables and analysis of variance tests for continuous normal variables. Biomarkers that were skewed were log_10_ transformed for analysis. Univariable and multivariable OR for mortality were estimated for each biomarker by logistic regression, comparing the top 3 quartiles to the lowest (reference) quartile. Maternal parity and marital status, and infant sex, baseline weight, and 6-week HIV RNA viral load were included as covariates, based on known risk factors for mortality [22]. Statistical analyses were performed using Stata version 10 (College Station, Texas) and Prism version 5 (GraphPad Software, La Jolla, California) software.

### Ethical Approvals

The ZVITAMBO trial and this substudy were approved by the Medical Research Council of Zimbabwe, Johns Hopkins Bloomberg School of Public Health Committee on Human Research, and Montreal General Hospital Ethics Committee.

## RESULTS

We included 663 infants; 272 were HIV infected (of whom 51 were selected at both time-points), 194 HIV exposed (6 selected at both time-points), and 197 HIV unexposed (3 selected at both time-points) (Figure 1).

### Impact of Biomarkers on Mortality in HIV-Infected Infants

We first evaluated associations between biomarkers and mortality among HIV-infected infants using a case-control design. HIV-infected infants who died in the first half of infancy (cases; n = 60), compared to HIV-infected infants who survived (controls; n = 120), had lower birth weight (mean [standard deviation {SD}], 2.77 [0.46] vs 2.92 [0.44] kg, respectively; *P* = .04) and 6-week weight (mean [SD], 4.10 [0.87] vs 4.50 [0.89] kg; *P* = .005), and were born to mothers with lower CD4 counts (mean [SD], 305 [150] vs 514 [223] cells/μL; *P* = .01) and higher viral loads (log mean [SD], 5.22 [0.65] vs 4.65 [0.66] copies/mL; *P* < .001) (Supplementary Table 1). Causes of death are shown in Supplementary Table 2. Cases, compared to controls, had similar 6-week concentrations of I-FABP and CRP, but marginally higher sCD14 (mean [SD], 1.48 [0.47] vs 1.34 [0.44] × 10^6^ pg/mL, respectively; *P* = .06) and IL-6 (median [interquartile range], 7.1 [4.6–10.0] vs 5.4 [3.7–7.8] pg/mL; *P* = .05). In a logistic regression model, OR for mortality were not significantly higher among infants with biomarker levels in the second, third, or fourth quartiles, compared to the first quartile, for any biomarker (Table 1). Similar results were obtained using biomarkers as continuous covariates in a logistic regression model, and when restricting cases to infants who died of pneumonia which was the predominant cause of death (data not shown).

**Table 1.** Odds Ratios for Mortality in Human Immunodeficiency Virus–Infected Infants by 6-Week Biomarker Levels

Biomarker	<25th Percentile (Ref)	25th–49th Percentile	50th–74th Percentile	≥75th Percentile
	OR	OR (95% CI)	OR (95% CI)	OR (95% CI)
I-FABP (58 cases/119 controls)^a^				
Unadjusted	1.0	0.8 (.3–1.8)	0.6 (.3–1.5)	0.6 (.2–1.4)
Adjusted^b^	1.0	1.6 (.6–4.3)	0.8 (.3–2.3)	0.8 (.3–2.1)
sCD14 (60 cases/120 controls)^a^				
Unadjusted	1.0	0.9 (.4–2.4)	3.0 (1.2–7.3)	1.9 (.8–4.8)
Adjusted^b^	1.0	0.7 (.3–2.2)	2.8 (1.0–8.0)	1.8 (.6–5.4)
IL-6 (45 cases/99 controls)^a^				
Unadjusted	1.0	1.3 (.5–3.9)	1.8 (.6–5.0)	2.5 (.9–7.0)
Adjusted^b^	1.0	0.8 (.2–2.9)	1.6 (.5–5.3)	2.0 (.6–6.3)
CRP (59 cases/120 controls)^a^				
Unadjusted	1.0	1.5 (.6–3.7)	1.2 (.5–3.1)	1.7 (.7–4.2)
Adjusted^b^	1.0	1.3 (.5–3.7)	1.3 (.5–3.8)	1.6 (.6–4.4)

Abbreviations: CI, confidence interval; CRP, C-reactive protein; I-FABP, intestinal fatty acid binding protein; IL-6, interleukin 6; OR, odds ratio; sCD14, soluble CD14.

^a^The number of cases and controls with available data is shown for each biomarker.

^b^Odds ratio for mortality between 6 weeks and 6 months of age based on quartiles of biomarkers measured in plasma at 6 weeks of age, adjusted for maternal parity and marital status, and infant sex, 6-week weight, and 6-week viral load in a logistic regression model.

HIV-infected infants who died in the second half of infancy (n = 23), compared to HIV-infected infants who survived (n = 120) had similar weight at birth and at 6 weeks, but significantly lower weight at 6 months (mean [SD], 5.87 [1.24] vs 6.77 [0.96] kg, respectively; *P* < .001). Mothers of cases and controls had no significant differences in baseline HIV disease status (CD4 count, viral load or mortality by 24 months postpartum) (Supplementary Table 3). Causes of death are shown in Supplementary Table 4. Cases compared to controls had similar 6-month levels of I-FABP and CRP, but significantly higher sCD14 (mean [SD], 2.12 [0.87] vs 1.76 [0.64] × 10^6^ pg/mL, respectively; *P* = .02) and IL-6 (median [interquartile range], 25.0 [13.9–28.8] vs 10.6 [7.1–17.1] pg/mL; *P* = .04). In a logistic regression model, the OR for mortality was not significantly higher among infants with biomarker levels in the second, third, or fourth quartiles, compared to the first quartile, for any biomarker (Table 2). Similar results were obtained using biomarkers as continuous covariates in a logistic regression model (data not shown).

**Table 2.** Odds Ratios for Mortality in Human Immunodeficiency Virus–Infected Infants by 6-Month Biomarker Levels

Biomarker	<25th Percentile (Ref)	25–49th Percentile	50th–74th Percentile	≥75th Percentile
	OR	OR (95% CI)	OR (95% CI)	OR (95% CI)
I-FABP (23 cases/108 controls)^a^				
Unadjusted	1.0	3.2 (.9–11.4)	2.0 (.5–7.4)	0.5 (.1–2.8)
Adjusted^b^	1.0	4.0 (.6–28.7)	2.5 (.3–22.6)	0.5 (.0–6.4)
sCD14 (23 cases/119 controls)^a^				
Unadjusted	1.0	3.7 (.9–14.9)	0.7 (.1–4.3)	3.8 (.9–15.5)
Adjusted^b^	1.0	1.2 (.2–8.9)	0.5 (.0–7.0)	2.3 (.4–14.6)
IL-6 (13 cases/60 controls)^a^				
Unadjusted	1.0	0.5 (.0–6.0)	1.7 (.2–11.6)	5.4 (.9–31.0)
Adjusted^b^	1.0	NA^c^	4.1 (.2–80.8)	2.1 (.1–43.1)
CRP (20 cases/108 controls)^a^				
Unadjusted	1.0	1.0 (.2–4.4)	2.0 (.5–7.5)	1.3 (.3–5.3)
Adjusted^b^	1.0	0.3 (.0–5.6)	1.6 (.2–12.5)	1.7 (.2–12.4)

Abbreviations: CI, confidence interval; CRP, C-reactive protein; I-FABP, intestinal fatty acid binding protein; IL-6, interleukin 6; NA, not applicable; OR, odds ratio; sCD14, soluble CD14.

^a^The number of cases and controls with available data is shown for each biomarker.

^b^Odds ratio for mortality between 6 and 12 months of age based on quartiles of biomarkers measured in plasma at 6 months of age, adjusted for maternal parity and marital status, and infant sex, 6-month weight, and 6-week viral load in a logistic regression model.

^c^Could not be estimated due to insufficient data.

### Biomarkers in HEU Infants

We next compared biomarkers between groups to test the hypothesis that HEU infants had higher levels of intestinal damage and inflammation than HIV-unexposed infants. Baseline characteristics of 6-week-old HIV-infected, HIV-exposed, and HIV-unexposed infants and their mothers are shown in Table 3. Infant groups were similar at birth, but mixed breastfeeding was more common among HIV-exposed infants and by 6 weeks there was a significant difference in weight between groups (*P* = .002). There was a significant difference among mothers in age, CD4 count, viral load, and mortality.

**Table 3.** Baseline Characteristics of 6-Week-Old Human Immunodeficiency Virus (HIV)–Infected, HIV-Exposed, and HIV-Unexposed Infants

Characteristic	HIV-Infected(n = 180)	HIV-Exposed(n = 100)	HIV-Unexposed(n = 100)	*P* Value^a^
Infant characteristics				
Male sex	87 (48.3)	50 (50.0)	51 (51.0)	.90
Delivery mode other than normal vaginal	16 (8.9)	10 (10.0)	5 (5.0)	.37
Birth weight, kg, mean (SD)	2.87 (0.45)	2.90 (0.44)	2.99 (0.46)	.10
Weight at 6 wk, kg, mean (SD)	4.37 (0.90)	4.47 (0.71)	4.74 (0.84)	.002
Method of feeding^b^				
Exclusive breastfeeding	7 (3.9)	7 (7.0)	7 (7.0)	.42
Predominant breastfeeding	41 (22.8)	20 (20.0)	26 (26.0)	.60
Mixed breastfeeding	93 (51.7)	70 (70.0)	56 (56.0)	.01
Maternal characteristics				
Age, y, mean (SD)	26.5 (5.5)	25.9 (5.1)	23.9 (5.2)	<.001
Married or stable union	161 (89.9)	87 (87.0)	96 (96.0)	.08
Parity, median (IQR)	2 (1–3)	2 (1–3)	2 (1–3)	.11
Education, y, mean (SD)	9.6 (2.4)	9.7 (2.2)	10.3 (1.8)	.06
Employed	22 (12.6)	13 (13.0)	13 (13.0)	.99
Family income, US$ per month, median (IQR)	77.3 (44.7–124.0)	78.9 (46.0–153.5)	70.9 (47.3–111.4)	.80
MUAC, cm, mean (SD)	25.5 (2.6)	25.5 (3.1)	25.9 (3.0)	.53
CD4 count, cells/μL, mean (SD) [No.]^c^	429 (220) [27]	523 (348) [16]	1010 (185) [7]	<.001
Viral load, log copies/mL, mean (SD) [No.]^c^	4.84 (0.70) [30]	4.21 (0.82) [16]	NA	.009
Mortality by 24 mo	12 (6.7)	7 (7.0)	0 (0.0)	.03

Data are presented as No. (%) unless otherwise indicated.

Abbreviations: HIV, human immunodeficiency virus; IQR, interquartile range; MUAC, mid-upper arm circumference; NA, not applicable; SD, standard deviation.

^a^Analysis of variance *P* value is shown for comparison between groups.

^b^Detailed feeding information was collected from mothers at 6 weeks, 3 months, and 6 months of age, including whether any of 22 liquids (water, juice, tea, cooking oil), milks (formula, fresh, tinned), medicines (traditional, oral rehydration solution, prescribed), or solid foods (porridge, sadza, fruit, vegetables, meat, eggs) had been given to the infant. Breastfeeding was defined as exclusive, predominant, or mixed at 3 months of age, according to previously published definitions [34]. Data on feeding were not available for 39 HIV-infected, 3 HIV-exposed, and 11 HIV-unexposed infants.

^c^Only measured in a subgroup of participants. Number of measurements for each group shown [No.].

**Figure 2.**
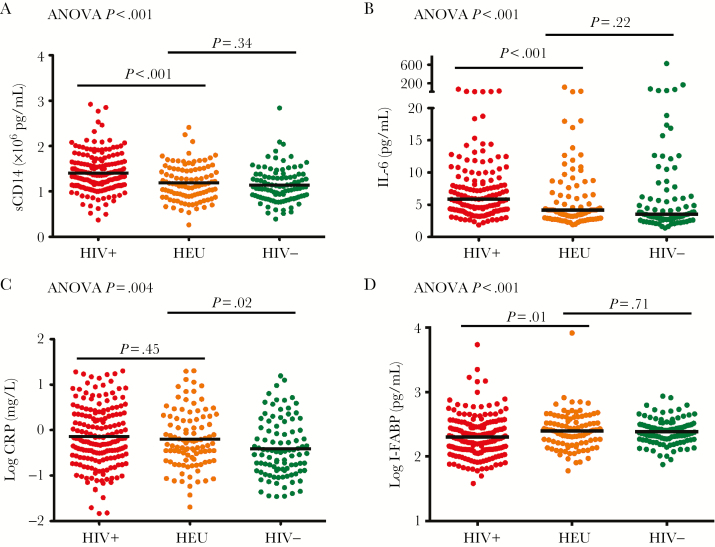
Biomarkers at 6 weeks of age. Concentrations of soluble CD14 (sCD14; *A*), interleukin 6 (IL-6; *B*); C-reactive protein (CRP; *C*), and intestinal fatty acid binding protein (I-FABP; *D*) at 6 weeks of age in human immunodeficiency virus (HIV)–infected (HIV^+^), HIV-exposed uninfected (HEU), and HIV-unexposed (HIV^–^) Zimbabwean infants. Analysis of variance (ANOVA) *P* value for the comparison of all 3 groups, and *P* values for comparisons between specific groups are shown.

Concentrations of all biomarkers at 6 weeks were significantly different between groups (Figure 2). Markers of monocyte activation (sCD14) and inflammation (IL-6) were highest in HIV-infected infants. Biomarker concentrations were generally similar between HIV-exposed and HIV-unexposed groups, except for CRP, which was significantly higher in HIV-exposed infants (log mean [SD], –0.20 [0.62] vs –0.41 [0.63] mg/L, respectively; *P* = .02) and not significantly different from levels in HIV-infected infants. I-FABP was higher in HIV-exposed and HIV-unexposed infants than in HIV-infected infants (log mean [SD], 2.40 [0.28] vs 2.39 [0.20] vs 2.30 [0.32] pg/mL, respectively; *P* < .001). Among HIV-infected infants, 6-week viral load correlated weakly with IL-6 (*R* = 0.18, *P* = .03, Spearman correlation) and sCD14 (*R* = 0.20, *P* = .007), but not with I-FABP or CRP.

Baseline characteristics of 6-month-old HIV-infected, HIV-exposed, and HIV-unexposed infants and their mothers are shown in Table 4. There was a significant difference between mothers in age, marital status, and parity, and HIV disease severity, as assessed by CD4 count and mortality. Infant groups were similar at birth, but by 6 weeks there was a significant difference in weight between groups (*P* = .004), which persisted to 6 months (*P* < .001).

**Table 4.** Baseline Characteristics of 6-Month-Old Human Immunodeficiency Virus (HIV)–Infected, HIV-Exposed, and HIV-Unexposed Infants

Characteristic	HIV-Infected(n = 143)	HIV-Exposed(n = 100)	HIV-Unexposed(n = 100)	*P* Value^a^
Infant characteristics				
Male sex	72 (50.4)	57 (57.0)	49 (49.5)	.50
Delivery mode other than normal vaginal	13 (9.1)	10 (10.0)	10 (10.0)	.96
Birth weight, kg, mean (SD)	2.88 (0.47)	3.01 (0.47)	2.97 (0.51)	.11
Weight at 6 wk, kg, mean (SD)	4.35 (0.80)	4.58 (0.72)	4.68 (0.79)	.004
Weight at 6 mo, kg, mean (SD)	6.63 (1.06)	7.36 (0.98)	7.41 (1.00)	<.001
Method of feeding^b^				
Exclusive breastfeeding	7 (4.9)	8 (8.0)	5 (5.0)	.55
Predominant breastfeeding	31 (21.7)	19 (19.0)	10 (10.0)	.06
Mixed breastfeeding	96 (67.1)	61 (61.0)	64 (64.0)	.61
Maternal characteristics				
Age, y, mean (SD)	26.6 (5.5)	26.5 (5.5)	24.6 (5.4)	.01
Married or stable union	128 (89.5)	98 (98.0)	96 (96.0)	.01
Parity, median (IQR)	2 (2–3)	2 (1–3)	2 (1–2)	.003
Education, y, mean (SD)	9.5 (2.3)	9.9 (2.0)	10.0 (2.3)	.14
Employed	19 (13.6)	15 (15.0)	16 (16.0)	.79
Family income, US$ per mo; median (IQR)	80.3 (44.7–138.3)	72.4 (51.2–158.6)	87.0 (50.0–153.7)	.52
MUAC, cm, mean (SD)	25.8 (2.6)	26.2 (3.2)	26.2 (3.3)	.50
Hemoglobin, g/L, mean (SD) [No.]^c^	115 (15) [14]	116 (14) [4]	128 (14) [9]	.13
CD4 count, cells/μL, mean (SD) [No.]^c^	532 (247) [21]	368 (216) [11]	930 (231) [6]	<.001
Viral load, log copies/mL, mean (SD) [No.]^c^	4.71 (0.67) [24]	4.51 (0.87) [14]	NA	.43
Mortality by 24 mo	2 (1.4)	7 (7.0)	0 (0.0)	.004

Data are presented as No. (%) unless otherwise indicated.

Abbreviations: HIV, human immunodeficiency virus; IQR, interquartile range; MUAC, mid-upper arm circumference; NA, not applicable; SD, standard deviation.

^a^Analysis of variance *P* value is shown.

^b^Detailed feeding information was collected from mothers at 6 weeks, 3 months, and 6 months of age, including whether any of 22 liquids (water, juice, tea, cooking oil), milks (formula, fresh, tinned), medicines (traditional, oral rehydration solution, prescribed) or solid foods (porridge, sadza, fruit, vegetables, meat, eggs) had been given to the infant. Breastfeeding was defined as exclusive, predominant or mixed at 3 months of age, according to previously published definitions [34]. Data on feeding were not available for 9 HIV-infected, 12 HIV-exposed, and 21 HIV-unexposed infants.

^c^Only measured in a subgroup of participants. Number of measurements for each group shown [No.].

**Figure 3.**
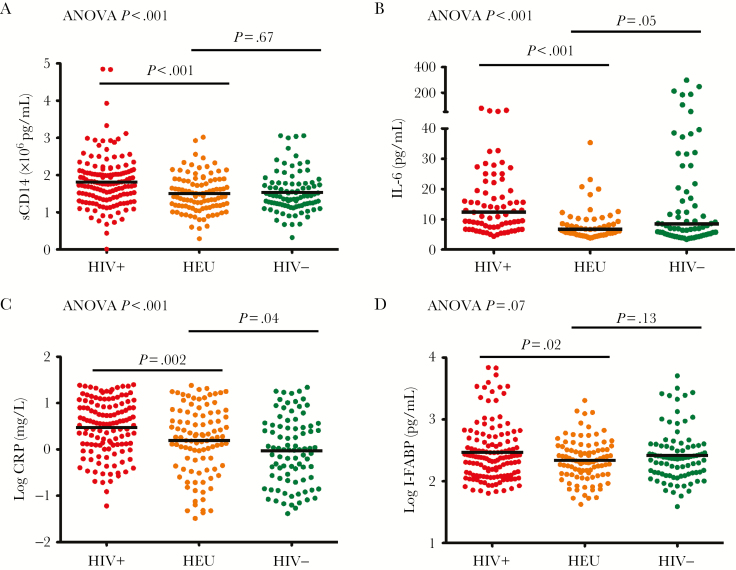
Biomarkers at 6 months of age. Concentrations of soluble CD14 (sCD14; *A*), interleukin 6 (IL-6; *B*), C-reactive protein (CRP; *C*), and intestinal fatty acid binding protein (I-FABP; *D*) at 6 months of age in human immunodeficiency virus (HIV)–infected (HIV^+^), HIV-exposed uninfected (HEU), and HIV-unexposed (HIV^–^) Zimbabwean infants. Analysis of variance (ANOVA) *P* value for the comparison of all 3 groups, and *P* values for comparisons between specific groups are shown.

Concentrations of inflammatory biomarkers at 6 months were significantly different between groups, with the highest levels in HIV-infected infants (Figure 3). Inflammatory biomarkers were higher across all infant groups at 6 months, compared to 6-week concentrations. Biomarker levels in HIV-exposed infants were generally similar to HIV-unexposed infants apart from CRP, which remained significantly higher (mean log [SD], 0.19 [0.73] vs –0.03 [0.73] mg/L, respectively; *P* = .04). I-FABP was significantly higher at 6 months than at 6 weeks among HIV-infected infants (*P* < .001), indicating increasing intestinal damage during infancy, whereas levels were not significantly higher by 6 months among the HIV-exposed and HIV-unexposed groups (*P* = .15 and *P* = .50, respectively). Levels of small-intestinal damage in HIV-infected infants at 6 months were significantly higher than in HIV-exposed infants (log mean [SD], 2.47 [0.46] vs 2.34 [0.33], respectively; *P* = .02).

### Impact of Biomarkers on HIV Transmission Through Breastfeeding

We finally tested the hypothesis that HIV-exposed infants acquiring HIV through breastfeeding had higher baseline levels of intestinal damage and inflammation than HEU infants.

HIV-exposed infants who acquired HIV through breastfeeding between 6 weeks and 6 months (n = 45), compared to HIV-exposed infants remaining uninfected by 6 months (n = 55), had lower birthweight (mean [SD], 2.78 [0.41] vs 3.02 [0.44] kg, respectively; *P* = .007) and 6-week weight (mean [SD], 4.21 [0.71] vs 4.68 [0.65] kg, *P* = .001) and were born to mothers with more advanced disease (mortality 13% vs 2%, *P* = .03). There were no differences in I-FABP at 6 weeks between infants who subsequently acquired or did not acquire HIV through breastfeeding (log mean [SD], 2.41 [0.32] vs 2.39 [0.25] pg/mL, respectively; *P* = .66). Infants who became infected had marginally higher baseline sCD14 (mean [SD], 1.26 [0.39] vs 1.13 [0.39] × 10^6^ pg/mL, respectively; *P* = .09) and significantly higher CRP (log mean [SD], –0.06 [0.63] vs –0.31 [0.60] mg/L; *P* = .04) than those remaining uninfected. However, after adjustment in a logistic regression model, there was no impact of any baseline biomarker on the odds of acquiring HIV (data not shown).

HIV-exposed infants who acquired HIV through breastfeeding between 6 and 12 months (n = 50), compared to HIV-exposed infants remaining uninfected through 12 months (n = 50), were similar in terms of maternal demographics and disease status, and had no significant differences in feeding pattern or weight through 6 months of age in this subsample. There were no differences in I-FABP concentrations between infants who subsequently acquired or did not acquire HIV through breastfeeding (log mean [SD], 2.31 [0.33] vs 2.36 [0.33] pg/mL, respectively; *P* = .48) and no difference in baseline levels of any inflammatory marker (data not shown). In a logistic regression model, there was no impact of any baseline biomarker on the odds of acquiring HIV (data not shown).

## DISCUSSION

This study evaluated biomarkers of intestinal damage and inflammation in Zimbabwean infants prior to cotrimoxazole and ART, and has 4 main findings. First, from 6 weeks of age, HIV-infected infants had high levels of inflammation, which further increased during infancy. Second, despite high levels of inflammation, no biomarker was associated with mortality, in contrast to older children and adults. Third, HEU infants had elevated CRP at 6 weeks and 6 months of age. Fourth, baseline levels of intestinal damage and inflammation among HIV-exposed infants were not related to breast-milk HIV acquisition.

Immune activation and CD4 decline are hallmarks of HIV infection; concentrations of inflammatory biomarkers are independently associated with mortality in HIV-infected adults [14–19] and older children [20]. Although elevated inflammatory markers have been reported in infancy [23–28], their relationship with mortality has not previously been evaluated. HIV-infected infants have rapid disease progression without ART; in this cohort, two-thirds died by 2 years [22]. Mortality was particularly high between 2 and 6 months of age [22], and our hypothesis was that inflammation prior to this mortality peak drives disease progression. Despite high levels of monocyte activation and inflammation—and higher levels of sCD14 and IL-6 among infants who died vs survived—we found no independent relationships between any biomarker and mortality. In a previous analysis, we found that the only independent predictors of mortality were maternal CD4 count and infant 6-week viral load [6]. We show here that inclusion of inflammatory biomarkers provides no additional discrimination in predicting mortality.

We found weak associations between infant viral load and inflammatory biomarkers, consistent with prior studies demonstrating that viral load is not strongly associated with immune activation in children [37, 38]. The causes of immune activation in pediatric HIV infection remain poorly characterized [39]. In adults, coinfections and microbial translocation are important [8]. We reasoned that intestinal damage, which is known to occur very early following adult HIV infection [9], may similarly occur in acute perinatal infection and allow microbial translocation. However, I-FABP levels at 6 weeks of age were unexpectedly lower in HIV-infected compared with HIV-exposed and HIV-unexposed infants. This may indicate that (*i*) small intestinal damage does not occur so soon after intrapartum infection; (*ii*) structural and immunological damage to the intestine is not reflected in circulating I-FABP levels [40]; (*iii*) other exposures are more critical determinants of I-FABP at a time of dynamic intestinal adaptation; or (*iv*) there is such extensive intestinal damage that enterocytes at the villus tip have been lost and are unable to release I-FABP, as in severe celiac disease [41]. By 6 months, I-FABP was clearly elevated in HIV-infected compared to HEU and HIV-unexposed infants, indicating that gut damage does occur in perinatal HIV infection; this is consistent with studies reporting elevated levels of lipopolysaccharide in HIV-infected infants by this age [24, 26, 28]. We were unable to measure lipopolysaccharide because samples were not collected into endotoxin-free tubes and we did not have other markers of microbial translocation; however, we found strikingly elevated sCD14 at both 6 weeks and 6 months in HIV-infected infants, consistent with prior infant studies [24, 26, 28]. Although sCD14 is not a specific marker of microbial translocation [42], it does reflect generalized monocyte activation [43].

Collectively, these findings indicate that immune activation is a cardinal feature of HIV infection in infants, as in older children and adults. We did not evaluate the causes of inflammation, but showed that intestinal damage increases over time in HIV-infected infants, consistent with prior studies [24, 26, 28]. However, levels of inflammatory biomarkers were not predictive of mortality. It is notable that sCD14 concentrations in infants who died were lower than among HIV-infected adults who died in the The Strategies for Management of Antiretroviral Therapy (SMART) trial (mean, 1.48 × 10^6^ pg/mL in infants vs 2.47 × 10^6^ pg/mL in adults) [36]. Drivers of inflammation may differ in infancy and not reflect pathogenic pathways underlying mortality; alternatively, inflammation may be an important driver of mortality but biomarker levels are insufficiently discriminatory to identify high-risk infants, similar to other prognostic markers in infancy [4, 7]. Regardless, chronic inflammation may be deleterious for growth and development and it is important to understand whether inflammation is normalized with early ART.

There is a growing recognition that HEU infants have higher morbidity and mortality than HIV-unexposed infants [29], and several immune abnormalities have been described [31]. We found that HEU infants had higher levels of inflammation than HIV-unexposed infants, as reported previously [44]. Here we extend these findings by showing that CRP concentrations in HEU infants at 6 weeks were similar to levels found in HIV-infected infants, and that CRP elevation persists at 6 months of age. We have previously speculated that inflammation may arise following exposure to HIV: a higher burden of coinfections; maternal enteropathy, and a distorted microbiota [45]. Future studies should evaluate the causes and consequences of this proinflammatory milieu and investigate whether inflammation resolves over time.

Postnatal HIV transmission remains a challenge in sub-Saharan Africa, where breastfeeding for 1–2 years is recommended. The mechanisms underlying breast-milk transmission are incompletely understood [30], though our trial previously demonstrated that exclusive breastfeeding was associated with reduced postnatal transmission [32]. In this study we hypothesized that higher levels of intestinal damage and inflammation may increase the risk of HIV acquisition, but we found no independent associations between baseline biomarker concentrations and HIV transmission throughout infancy. A previous study of Malawian infants [24] similarly found that baseline immune activation was not associated with HIV acquisition, but higher levels of lipopolysaccharide increased the risk of breast-milk transmission, suggesting that intestinal barrier function may be critical. We did not have markers of intestinal permeability in our study; although I-FABP levels reflect small intestinal villous damage [41], they do not necessarily reflect mucosal integrity.

This study had strengths and weaknesses. We took advantage of a birth cohort with well-characterized HIV exposure and infection status and robust mortality ascertainment prior to cotrimoxazole and ART, enabling a “natural history” study of mortality and breast-milk HIV transmission using available specimens; however, we only had plasma samples, which restricted the assays we could conduct. We only measured biomarker concentrations at 2 time-points and may have missed critical periods of inflammation. Although we had sufficient cases and controls in the first half of infancy, there were few deaths between 6 and 12 months of age, meaning that our study may have been underpowered to evaluate mortality later in infancy.

In summary, we show that infants acquiring HIV at birth have inflammation as early as 6 weeks of age, which increases over time and is associated with evolving intestinal damage, similar to adult HIV infection; however, in contrast to adults and older children, we found no associations with mortality. HIV-exposed but uninfected infants have ongoing inflammation until at least 6 months of age, which may contribute to poor health outcomes. Finally, the extent of enterocyte damage and systemic inflammation was not a risk factor for breast-milk HIV transmission in infancy. These findings extend our understanding of the similarities and differences between HIV infection in infancy and later life and highlight areas for further investigation.

## Supplementary Data

Supplementary materials are available at *The Journal of Infectious Diseases* online. Consisting of data provided by the authors to benefit the reader, the posted materials are not copyedited and are the sole responsibility of the authors, so questions or comments should be addressed to the corresponding author.

## Supplementary Material

Supplementary Tables

## Notes

***Financial support.*** This study was funded by the Wellcome Trust (grant number 093768/Z/10/Z). The ZVITAMBO trial was supported by the Canadian International Development Agency (R/C Project 690/M3688); the US Agency for International Development (USAID) (cooperative agreement number HRN-A-00-97-00015-00 between Johns Hopkins University and the Office of Health and Nutrition–USAID); and a grant from the Bill & Melinda Gates Foundation (Seattle, Washington). Additional funding was received from the SARA Project, which is operated by the Academy for Educational Development, Washington, DC, and is funded by USAID’s Bureau for Africa, Office of Sustainable Development, under the terms of contract AOT-C-00-99-00237-00, the Rockefeller Foundation (New York, New York), and BASF (Ludwigshafen, Germany).

***Potential conflicts of interest.*** All authors: No potential conflicts of interest. All authors have submitted the ICMJE Form for Disclosure of Potential Conflicts of Interest. Conflicts that the editors consider relevant to the content of the manuscript have been disclosed.
