# Supplementary material for: Intestinal Damage and Inflammatory Biomarkers in Human Immunodeficiency Virus (HIV)–Exposed and HIV-Infected Zimbabwean Infants
Source: J Infect Dis. 2017 Jul 28;216(6):651–61. doi: 10.1093/infdis/jix367 (PMC5853317; doi:10.1093/infdis/jix367)
Supplement: Supplementary Tables [file jix367_suppl_supplementary_tables.docx]

**Intestinal damage and inflammatory biomarkers in HIV-exposed and HIV-infected Zimbabwean infants – Supplementary Material**

**CONTENTS**

**Supplementary Table 1 – Characteristics of HIV-infected cases and controls at 6 weeks of age Pages 2-3**

**Supplementary Table 2 – Causes of death between 6 weeks and 6 months of age in HIV-infected infants Page 4**

**Supplementary Table 3 – Characteristics of HIV-infected cases and controls at 6 months of age Pages 5-6**

**Supplementary Table 4 – Causes of death between 6 and 12 months of age in HIV-infected infants Page 7**

**Supplementary Table 1 – Characteristics of HIV-infected cases and controls at 6 weeks of age**

**Characteristic Cases^a^ Controls^a^ P value**

**(N=60) (N=120)**

**Infant characteristics**

Male; % (n) 50.0 (30) 47.5 (57) 0.75

Delivery mode other than normal vaginal; % (n) 5.0 (3) 10.8 (13) 0.21

Birth weight, kg; mean (SD) 2.77 (0.46) 2.92 (0.44) 0.04

Weight at 6 weeks, kg; mean (SD) 4.10 (0.87) 4.50 (0.89) 0.005

Haemoglobin at 6 weeks, g/L; mean (SD) [n]^b^ 107 (18) [19] 101 (23) [45] 0.34

Viral load at 6 weeks, log copies/mL; mean (SD) [n]^b^ 5.01 (0.65) [56] 4.60 (0.71) [114] <0.001

**Maternal characteristics**

Age, years; mean (SD) 26.3 (5.7) 26.5 (5.4) 0.82

Married or stable union; % (n) 96.6 (57) 86.7 (104) 0.04

Parity; median (IQR) 2 (1, 3) 2 (1, 3) 0.54

Education, years; mean (SD) 9.8 (2.5) 9.5 (2.4) 0.46

Employed; % (n) 17.2 (10) 10.3 (12) 0.19

Family income, US$ per month; median (IQR) 94.6 (47.6, 165.9) 73.2 (36.6, 113.5) 0.15

MUAC, cm; mean (SD) 25.5 (2.9) 25.5 (2.5) 0.92

Haemoglobin, g/L; mean (SD)[n]^b^ 109 (20) [5] 111 (18) [13] 0.78

CD4 count, cells/uL; mean (SD) [n]^b^ 305 (150) [11] 514 (223) [16] 0.01

Viral load, log copies/mL; mean (SD) [n]^b^ 5.22 (0.65) [10] 4.65 (0.66) [20] <0.001

Mortality by 24 months; % (n) 8.3 (5) 5.8 (7) 0.53

^a^Cases were intrapartum HIV-infected infants who died between 6 weeks and 6 months of age. Controls were intrapartum HIV-infected infants who survived between 6 weeks and 6 months of age.

^b^Only measured in a subgroup of participants. Number of measurements for each group shown [n].

MUAC: Mid-upper arm circumference

**Supplementary Table 2 – Causes of death between 6 weeks and 6 months of age in HIV-infected infants**

**Cause of death^a^ N**

Pneumonia 39

Pneumonia and diarrhoea 4

Pneumonia and malnutrition 3

Pneumonia and meningitis 1

Pneumonia and tuberculosis 1

Uncertain 11

Diarrhoea 1

**Total 60**

^a^Causes of death were assigned by a study paediatrician, blinded to maternal and infant HIV status. Multiple causes were allowed and were not ranked. If available, the primary source of cause of death was records from the hospital admission during which the child died, or one near to the time of death. When hospital records were not available, cause of death was assigned based on verbal autopsy data, as described in [22].

**Supplementary Table 3 – Characteristics of HIV-infected cases and controls at 6 months of age**

**Characteristic Cases^a^ Controls^a^ P value**

**(N=23) (N=120)**

**Infant characteristics**

Male; % (n) 52.2 (12) 50.0 (60) 0.85

Delivery mode other than normal vaginal; % (n) 8.7 (2) 9.2 (11) 0.94

Birth weight, kg; mean (SD) 2.81 (0.53) 2.89 (0.46) 0.46

Weight at 6 weeks, kg; mean (SD) 4.29 (0.81) 4.36 (0.79) 0.72

Weight at 6 months, kg; mean (SD) 5.87 (1.24) 6.77 (0.96) <0.001

Haemoglobin at 6 weeks, g/L; mean (SD) [n]^b^ 109 (20) [10] 109 (23) [55] 0.98

Viral load at 6 weeks, log copies/mL; mean (SD) [n]^b^ 4.69 (0.74) [11] 4.40 (0.77) [85] 0.25

**Maternal characteristics**

Age, years; mean (SD) 26.3 (5.7) 26.5 (5.4) 0.82

Married or stable union; % (n) 96.6 (57) 86.7 (104) 0.04

Parity; median (IQR) 2 (1, 3) 2 (1, 3) 0.54

Education, years; mean (SD) 9.8 (2.5) 9.5 (2.4) 0.46

Employed; % (n) 18.6 (11) 11.8 (14) 0.19

Family income, US$ per month; median (IQR) 65.1 (34.7, 97.4) 81.2 (47.3, 138.6) 0.21

MUAC, cm; mean (SD) 25.5 (2.9) 25.5 (2.5) 0.92

Haemoglobin at 6 weeks, g/L; mean (SD) [n]^b^ 115 (7) [2] 115 (16) [12] 0.99

CD4 count, cells/uL; mean (SD) [n]^b^ 562 (235) [3] 527 (255) [18] 0.83

Viral load at 6 weeks, log copies/mL; mean (SD) [n]^b^ 4.59 (0.24) [4] 4.73 (0.73) [20] 0.71

Mortality by 24 months; % (n) 4.4 (1) 0.8 (1) 0.19

^a^Cases were intrapartum HIV-infected infants who died between 6 and 12 months of age. Controls were intrapartum HIV-infected infants who survived between 6 and 12 months of age.

^b^Only measured in a subgroup of participants. Number of measurements for each group shown [n].

MUAC: Mid-upper arm circumference

**Supplementary Table 4 – Causes of death between 6 and 12 months of age in HIV-infected infants**

**Cause of death^a^ N**

Pneumonia 6

Pneumonia and diarrhoea 6

Pneumonia and malnutrition 1

Pneumonia and meningitis 1

Pneumonia and tuberculosis 1

Uncertain/other 5

Diarrhoea 1

Diarrhoea and malnutrition 1

Meningitis 1

**Total 23**

^a^Causes of death were assigned by a study paediatrician, blinded to maternal and infant HIV status. Multiple causes were allowed and were not ranked. If available, the primary source of cause of death was records from the hospital admission during which the child died, or one near to the time of death. When hospital records were not available, cause of death was assigned based on verbal autopsy data, as described in [22].
